# Exploring approaches for predictive cancer patient digital twins: Opportunities for collaboration and innovation

**DOI:** 10.3389/fdgth.2022.1007784

**Published:** 2022-10-06

**Authors:** Eric A. Stahlberg, Mohamed Abdel-Rahman, Boris Aguilar, Alireza Asadpoure, Robert A. Beckman, Lynn L. Borkon, Jeffrey N. Bryan, Colleen M. Cebulla, Young Hwan Chang, Ansu Chatterjee, Jun Deng, Sepideh Dolatshahi, Olivier Gevaert, Emily J. Greenspan, Wenrui Hao, Tina Hernandez-Boussard, Pamela R. Jackson, Marieke Kuijjer, Adrian Lee, Paul Macklin, Subha Madhavan, Matthew D. McCoy, Navid Mohammad Mirzaei, Talayeh Razzaghi, Heber L. Rocha, Leili Shahriyari, Ilya Shmulevich, Daniel G. Stover, Yi Sun, Tanveer Syeda-Mahmood, Jinhua Wang, Qi Wang, Ioannis Zervantonakis

**Affiliations:** ^1^ Cancer Data Science Initiatives, Frederick National Laboratory for Cancer Research, Frederick, MD, United States; ^2^ Department of Ophthalmology and Visual Sciences, The Ohio State University Wexner Medical Center and James Comprehensive Cancer Center, Columbus, OH, United States; ^3^ Institute for Systems Biology, Seattle, WA, United States; ^4^ Department of Civil and Environmental Engineering, University of Massachusetts Amherst, Amherst, MA, United States; ^5^ Innovation Center for Biomedical Informatics, Georgetown University, Washington DC, United States; ^6^ Department of Veterinary Medicine and Surgery, University of Missouri, Columbia, MO, United States; ^7^ Department of Biomedical Engineering and OHSU Center for Spatial Systems Biomedicine (OCSSB), Oregon Health and Science University, Portland, OR, United States; ^8^ School of Statistics, University of Minnesota, Minneapolis, MN, United States; ^9^ Department of Therapeutic Radiology, Yale University School of Medicine, Yale University, New Haven, CT, United States; ^10^ Department of Biomedical Engineering, University of Virginia, Charlottesville VA, United States; ^11^ Stanford Center for Biomedical Informatics Research (BMIR), Department of Medicine and Department of Biomedical Data Science, Stanford University, Stanford, CA, United States; ^12^ Center for Biomedical Informatics and Information Technology, National Cancer Institute, National Institutes of Health, Bethesda, MD, United States; ^13^ Department of Mathematics, The Pennsylvania State University, University Park, PA, United States; ^14^ Mathematical NeuroOncology Lab, Precision Neurotherapeutics Innovation Program, Mayo Clinic Arizona, Phoenix, AZ, United States; ^15^ Computational Biology and Systems Medicine Group, Centre for Molecular Medicine Norway University of Oslo, Oslo, Norway; ^16^ Department of Pharmacology and Chemical Biology, University of Pittsburgh, Pittsburgh, PA, United States; ^17^ Department of Intelligent Systems Engineering, Indiana University, Bloomington, IN, United States; ^18^ Department of Mathematics and Statistics, University of Massachusetts Amherst, Amherst, MA, United States; ^19^ School of Industrial and Systems Engineering, The University of Oklahoma, Norman, OK, United States; ^20^ Division of Medical Oncology and Department of Medicine, The Ohio State University Comprehensive Cancer Center, Columbus, OH, United States; ^21^ Department of Mathematics, University of South Carolina, Columbia, SC, United States; ^22^ Almaden Research Center, IBM Research, San Jose, CA, United States; ^23^ Institute for Health Informatics and the Masonic Cancer Center, University of Minnesota, Minneapolis, MN, United States; ^24^ Department of Bioengineering, UPMC Hillman Cancer Center, University of Pittsburgh, Pittsburgh, PA, United States

**Keywords:** digital twins, oncology, cancer patient, predictive medicine, artificial intelligence, mathematical modeling, machine learning

## Abstract

We are rapidly approaching a future in which cancer patient digital twins will reach their potential to predict cancer prevention, diagnosis, and treatment in individual patients. This will be realized based on advances in high performance computing, computational modeling, and an expanding repertoire of observational data across multiple scales and modalities. In 2020, the US National Cancer Institute, and the US Department of Energy, through a trans-disciplinary research community at the intersection of advanced computing and cancer research, initiated team science collaborative projects to explore the development and implementation of predictive Cancer Patient Digital Twins. Several diverse pilot projects were launched to provide key insights into important features of this emerging landscape and to determine the requirements for the development and adoption of cancer patient digital twins. Projects included exploring approaches to using a large cohort of digital twins to perform deep phenotyping and plan treatments at the individual level, prototyping self-learning digital twin platforms, using adaptive digital twin approaches to monitor treatment response and resistance, developing methods to integrate and fuse data and observations across multiple scales, and personalizing treatment based on cancer type. Collectively these efforts have yielded increased insights into the opportunities and challenges facing cancer patient digital twin approaches and helped define a path forward. Given the rapidly growing interest in patient digital twins, this manuscript provides a valuable early progress report of several CPDT pilot projects commenced in common, their overall aims, early progress, lessons learned and future directions that will increasingly involve the broader research community.

## Introduction

A paradigm shift appears underway in the use of digital twin approaches to advance new methods for precision and predictive cancer care. This shift is motivated not only to better diagnose and treat the individual, but also from the point of view of transforming care by more effectively involving the individual patient in their health and care decisions over their lifetime (1). Cancer is not a single disease, but a family of diseases that share certain common characteristics resulting in uncontrolled cellular proliferation and destructive invasion of tissue (2). The complexity of cancer extends beyond the individual cancer cells to include the “normal” cells in the tumor environment and the individual as a whole; the unique underlying genomics and functional systems of the body are critical in the response to both disease and treatments. Precision oncology, according to the Precision Medicine Initiative^1^ aims to diagnose and treat patients with increased specificity based on a more in-depth and precise understanding of an individual's disease, taking into account the added scientific insights, similarities, and differences among patients, many of these gained from multiomic analyses.

A digital twin, as defined by the Digital Twin Consortium, is a virtual embodiment of a real-world object or system, historically based and continuously updated to mirror the behavior of the object in the real world^2^. Digital twins have been used for several years in multiple industries to predict behavior, monitor an object's activities and responses, and predict potential future situations that would support decisions to initiate preemptive maintenance or guide different behavior. As real-world systems are complex, so too are digital twins created at different levels of detail, at multiple scales, and represent many different objects and/or systems. After insightfully detailing how digital twins have been used in industry, Croatti et al. (3) recently posited that digital twins can be applied to healthcare systems, thus providing an approach to monitor and manage the multiple interacting components in the healthcare system while incorporating such emerging technologies as the Internet of Things (IoT) to support real-time data acquisition. Fertig et al. (4) elaborated further on how data assimilation methods from weather forecasting can be adapted to cancer forecasting, particularly when patient models merge domain knowledge with data-driven insights into rigorous computational models. More recently, and aligned with the work of this manuscript, Wu et al. (5) described digital twins and proposed a framework to extend the concept of digital twins to individual patients, providing insights into opportunities and challenges facing imaging-driven digital twin approaches. The concept of a cancer patient digital twin (CPDT) continues to gain interest and inspire the cancer community, as shared in a recent report exploring the future for predictive radiation oncology (6).

## Background and motivation

The concept for the CPDT efforts described in this paper has its origin in the NCI-DOE Collaboration, established in 2016 (7). In the wake of the Precision Medicine Initiative (referenced previously) and the emergence of the National Strategic Computing Initiative^3^, this unique collaboration set forth with aims to pursue cancer challenges that could leverage predictive modeling, simulations, and AI to make significant progress, while at the same time informing the development of exascale computing co-design through the application to large-scale biological challenges. As part of the first Cancer Moonshot^SM^ effort, three pilot projects were pursued with aims to develop new capabilities at the forefront of multi-scale molecular scale predictive modeling (8), predicting tumor response to drug treatments (9, 10) and monitor cancer patients more efficiently and effectively using natural language processing of cancer pathology reports (11, 12). Indeed, these pilot project efforts each pushed the frontiers for emerging technologies needed to develop cancer patient digital twins including multi-scale modeling, cancer patient surveillance and health trajectories, and prediction of cancer treatment outcomes.

With the groundwork laid by the NCI-DOE pilot projects and continuing efforts to grow the trans-disciplinary community while pushing the frontiers of computing and cancer, the concept of digital twins and cancer patients converged at the 2017 Frontiers of Predictive Oncology and Computing meeting,^4^ and parallels were identified between industry successes in digital twin approaches and employing the latest advances involving large scale DOE computing to advance cancer research. During the ensuing 2019 Envisioning Computational Innovations for Cancer Challenges meeting, the cancer patient digital twin concept was further developed, establishing the foundation for pursuing the envisioned CPDT. The vision is for the CPDT to build on the new NCI-DOE capabilities being developed and provide a driving shared community goal that would foster broad community involvement and advance new modeling approaches to a level of scientific, computational, data, and community integration needed to cross the barrier to an integrated cancer patient digital twin (1).

Building on the need for integrated and cross-disciplinary team science, the exploratory projects for the CPDT described below were developed using a cross-organization and interdisciplinary methodology which involved having 30 researchers from 25 organizations across multiple disciplines come together for a weeklong Ideas Lab^5^ in 2020 to develop the exploratory CPDT projects described below. Participants self-organized around common areas of interest in CPDT approaches and worked together with experienced mentors to define shared project plans that established a clear conceptual vision and technical roadmap for respective CPDT approaches. After internal NCI and DOE review, the following five projects described below were selected for short-term funding to develop the concepts and approaches for advancing cancer patient digital twins.

## Cancer patient digital twin exploratory projects

### Project 1: simulating one million pancreatic cancer patients to guide treatment

#### Project description: lead institution—Georgetown University

The initial goal of this project was to develop methods that connect the progression, therapeutic interventions, and outcomes from a cohort of pancreatic cancer patients to simulation results generated from a model of subclonal tumor evolution. The model tracked the growth of 4 subclone populations as they were subjected to treatment with one of any two, non-cross resistant therapies. In terms of these two interventions, subclones represent cell populations that are sensitive to both, resistant to the first, resistant to the second, and resistant to both. Transitions of individual cells between sensitive to resistant populations occur according to characteristic subclonal evolutionary rates. Every 45 days, the model will apply one of the two therapies, or both in combination at a reduced dose, and compare different strategies to make that choice.

The simulation results represented a pan-cancer analysis of a parameter space spanning the range of realistic values that define subclonal sensitivity to therapeutic intervention and the kinetics of growth and emergence of drug resistance. *In silico*, the evolutionary model doubles the survival of virtual patients on average (13, 14). The first two 45-day periods are particularly critical in achieving these results (15). The team had extensive data describing the patient demographics, molecular profiling identifying actionable biomarkers, and the ordering and duration of therapeutic interventions in a population of pancreatic cancer patients. However, matching real patients in this cohort with simulated patients from the model did not result in a definitive mapping between real-world data and model parameters. The patient data lacked the temporal resolution to profile tumor response to therapeutic interventions, and a population-based approach that mapped patients with similar biomarkers and treatment schedules to simulation outcomes did not uniquely converge in the model parameter space.

#### Observations and future efforts

While the work did not result in a mapping algorithm between patient data and model input parameters, the process defined some very important challenges to utilizing CPDTs for guiding precision therapy. Patient data are, and will continue to be, incomplete, especially with respect to the resolution needed to uniquely identify any single model parameter. At best, the team can estimate the confidence in a specific patient-mapped input parameter and translate that to a range around the associated value. Through the combinatorial expansion of values that span the range of possible values, a single patient is then represented by a population CPDTs. Even for models with a modest number of adjustable parameters, this population will be quite large.

Depending on how heterogeneous the simulation outcomes are, the challenge then becomes identifying which of the CPDTs in the population are truly representative of the given patient. Results can be used to identify population subsets with divergent responses to a given therapy, and analysis could then inform subsequent data collection. Consider the case in which a majority of the patient-specific CPDTs are predicted to have a similar response to different therapies, but there is a subset of the population with a drastically improved outcome with one intervention with respect to the other. Understanding what combinations of parameter values led to the divergent model behavior could direct additional data collection that would definitively place the patient in one of the subsets of their CPDT population. The challenge is finding the least invasive way to provide the highest resolution data collection possible.

For any model as an approximation of a complex system, it is expectedly impossible to exactly match patient data to any model parameter; there will always be some error or uncertainty. Practically, this means data from a single patient will result in a population of CPDTs, likely with divergent outcomes for any of the tested intervention strategies. The challenge will then become how to collect additional data that will refine the population into an increasingly accurate representation of the individual's therapeutic response. Project future work aims to understand the regions of the complex parameter space for which high-resolution measurement is needed, and to deconvolute their relationships to CPDT simulation output.

### Project 2: self-learning platforms for personalized treatment of melanoma

#### Project overview: lead institution—Indiana University

**Figure 1:**
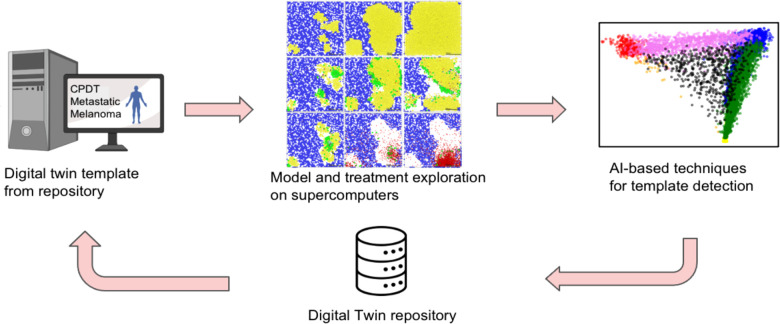
Overall approach to identify digital patient templates.

This project is an initial stage of an aspirational vision to develop clinically actionable CPDTs for planning immunotherapy in metastatic melanoma patients. The team leveraged cutting-edge multiscale models of heterogeneous tumors and immune system dynamics to create multiscale models of tumor-immune interactions in melanoma pulmonary metastases, aided by canine data to drive rapid model refinement. HPC-driven model exploration will ensure that the multiscale model can recapitulate essential clinical trajectories including spontaneous regression, arrest at sub-clinical size, and growth to clinical detection. Techniques grounded in Artificial Intelligence (AI) are used to analyze the simulated patient trajectories and develop CPDT templates—a key step in fitting models to individual patients (see Figure 1).

The overarching goal is to develop and implement a model of metastatic melanoma for autologous cancer vaccine immunotherapies and to prepare for prototyping and testing against canine data. To reach this goal, the pilot project aims to (1) construct a multiscale model of melanoma metastases, (2) perform model exploration on HPC to identify digital patient templates (3) extend the model to autologous vaccine immunotherapy, (4) prepare sample longitudinal canine data for framework testing, and (5) perform initial (human) clinical case selection.

#### Observations and future efforts

Building upon work by Macklin's COVID-19 modeling coalition (16), the team designed a multiscale agent-based model of melanoma pulmonary micrometastases with local and systems-scale immune interactions, including innate and adaptive immune responses to the infiltrating cancer cells. The team verified that the model could capture clinically salient outcomes, including uncontrolled growth, partial tumor control, and complete tumor elimination. The team also varied and explored ten key immune system parameters on HPC resources and applied AI techniques to the simulation data to analyze the digital patient trajectories. The significant heterogeneity in the trajectories required novel clustering methods to identify patient templates. The team also found that it was possible to mimic vaccine immunotherapy by introducing virtual tumor cell debris to prime immune interactions.

For future model training, the team selected and annotated four canine melanoma cases, including clinical findings, weight, primary and metastasis measurements, mitosis, and immune cell infiltration. The team also identified 9,295 melanoma patients from the Stanford Comprehensive Cancer Center with diverse gender, ethnicity, age, and treatment characteristics. The team is currently analyzing these trajectories to retrospectively identify optimal treatment patterns.

Multiscale CPDTs (with many parameters) are challenging to fit to individual patients with routine clinical measurements. CPDT templates address this challenge by reducing the size of the “search space” for model fitting, and data assimilation techniques can further tailor the templates to individuals throughout their treatment. Over time, an accumulated repository of fitted CPDTs can help refine the templates, effectively transferring knowledge from prior patients to new patients. Because the models incorporate fundamental cell behaviors, they can be mechanistically updated to incorporate new biological discoveries. The open framework approach encourages community contributions to these model components, allowing the community to pool resources, combine expertise, and more rapidly advance towards CPDTs that improve patient outcomes and quality of life.

### Project 3: an adaptive digital twin approach for monitoring treatment response and resistance

#### Project overview: lead institution—Stanford University

**Figure 2:**
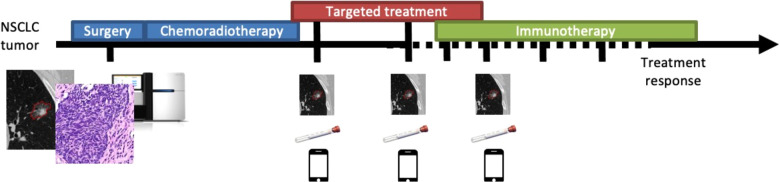
Longitudinal and multi-modal data capture for a lung cancer patient: timeline of a composite lung cancer patient showing possible treatment strategies and possible biomedical data modalities.

In this project, the team is developing a CPDT by integrating baseline multi-modal data and repeated measurements for real-time dynamic model training and updating (17). The approach leverages innovations in the areas of cancer research, artificial intelligence, and computing technologies to realize a CPDT for monitoring treatment response and treatment resistance (18). Specifically, the effort proposes an adaptive dynamic CPDT, using baseline features to predict response to therapy, enabling treatment reassignment should predictions not meet expectations (Figure 2). During the maintenance phase, the CPDT will help additionally assess resistance mechanisms and similarly enable effective treatment reassignment. This CPDT will help physicians make initial treatment determinations, comparatively monitor treatment response, assess toxicity and effectiveness, and decide when to discontinue or change an approach.

The team is developing initial modules for each of the data modalities proposed as essential components of the lung cancer digital twin approach: demographic data, CT imaging, digital pathology, histopathologic data, and genomics. The team is curating a retrospective dataset that will serve as one of the main cohorts to develop, fine-tune and test this lung cancer digital twin (19). Next the team will build deep learning models to integrate these data modalities (20) into an adaptive predictive CPDT model.

#### Observations and future efforts

The team has built a lung lesion variational auto-encoder that can successfully reconstruct 3D volumes of lung nodules and yield meaningful embeddings of the nodules. Such models are shown to have the ability to predict tumor volume and could potentially be used for other downstream task predictions including genomic features such as EGFR mutation status (21, 22). On top of that, the model can easily generalize to other lung CT datasets without any fine-tuning and can predict tumor volume changes with similar qualities as the dataset it was trained on (manuscript in preparation). Secondly, the team has developed a variational autoencoder (VAE) that learns a latent representation of lung tissue gene expression profiles and has early observations that this model is able to generate realistic synthetic gene expression. This representation is used to infuse generative adversarial networks (GAN), generating lung tissue tiles with a new model that we call RNA-GAN.

When model training is faced with small datasets or datasets, missing modalities, or studies in which multi-modal data are expensive to produce, or especially for multi-modal problems, early results show the promise of multi-modal biological data imputation and obtain better quality multi-modal data. In future work, the team is working to connect each of the modules just described to eventually develop a multiscale model of a lung cancer lesion. The approach employs a late fusion strategy to bring all modules together and predict tumor size in the context of two treatment modalities: anti-EGFR treatment and immunotherapy. The team will also investigate the estimation of the cellular microenvironment of lung cancer patients, and its use to determine tumor evolution. One of the main challenges to continuing the work on a lung cancer DT will be to collect and share multi-institutional multi-modal longitudinal cohorts of lung cancer patients in a privacy-preserving manner. Even though public databases are available, most publicly available cohorts are not multimodal and not longitudinal. A federated learning approach may offer a solution to share multi-modal longitudinal biomedical data more seamlessly without the need for data sharing agreements.

### Project 4: a patient-specific multiscale digital twin for the exploration of optimal treatment pathways for non-small cell lung cancer

#### Project overview: lead institution—University of South Carolina

This project aims to develop a dynamic, multiscale cancer patient digital twin (CPDT) for a non-small cell lung cancer (NSCLC) patient by harnessing the patient's own medical data and leveraging data from similar patients in the population. It will be deployed to search for optimal pathways for the specific cancer patient by exploring the treatment pathway space dynamically. The digital twin by design is a virtual replica of the NSCLC patient, recording the patient's past state, monitoring the patient's present state, and forecasting the patient's future state.

The team takes an *in silico* approach to build the dynamic, multiscale CPDT leveraging an HPC infrastructure, exploiting multimodal patient data across observational and treatment scales longitudinally, as well as cross-sectionally, as an initial digital twin. The team then leverages data from similar patients in the population to simulate various nuanced treatment choices in a treatment pathway graph to form clinical recommendations. The CPDT leverages four key sources of information and *in silico* tools: (1) medical data, including diagnostic notes, of the cancer patient and longitudinal data from similar patients; (2) *a priori* clinical knowledge of existing treatment pathways from various institutions; (3) prior treatment pathways of existing patients from electronic health records; and (4) simulations of treatment pathways exploring patient similarity. The CPDT will adopt the latest advances in artificial intelligence (AI), especially, deep learning models and technologies, and physics-informed modeling and simulations.

**Figure 3:**
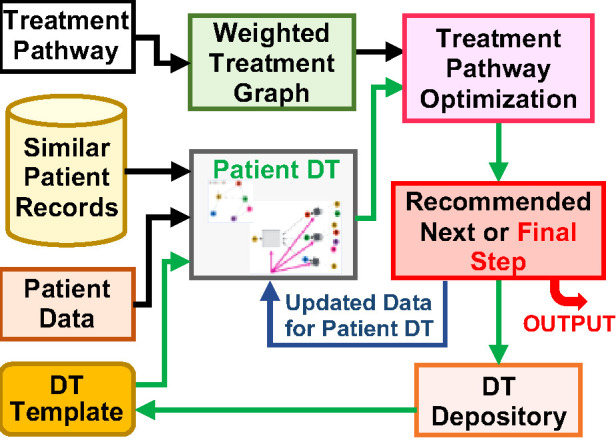
The schematic of a multiscale digital twin for a lung cancer patient.

To build the CPDT, the team leverages the existing NSCLC patient data in available databases and in-house datasets. This is a large collaborative endeavor across several traditionally orthogonal disciplines, requiring complementary expertise working seamlessly on multiscale mechanistic modeling and implementation, regulatory protein network development, patient pathway infrastructure development, data fusion technologies, and trustworthy AI. New AI-enabled mechanical and mechanistic models, latent-space representations, multigraph neural networks for the fusion of multiple modalities, and evolutionary models will be innovated by exploiting the hidden information in the dynamical, longitudinal dataset of the patient. A novel computational platform integrating mechanical/physical models and machine learning will be developed leveraging the advanced computational capability in HPC environments. A schematic of the basic components in a multiscale DT is shown in Figure 3 above.

#### Observations and future efforts

The team will actively engage in the digital twin development community and work closely with the other developers to improve and update our design and implementation. The digital twin model will be developed following the state-of-the-art trustworthy AI criteria. Its fundamental architecture will be made extensible and adaptable to other medical applications through enhanced transfer learning. A rigorous model assessment procedure will be implemented to ensure the safe and effective use of the model in clinical settings. So far, the team has gathered a set of NSCLC patient data for a cohort of patients treated at Yale-New Haven Health System and from publicly available databases, made progress on the physiological module in the multiscale DT (23), developed deep learning guided similarity analysis of patients (24, 25), and started building a portion of protein network dynamics. Various deep learning tools including LSTM and more general neural dynamical systems have been implemented on time-series data of metabolic panels of cancer patients. Transfer learning has been adopted to translate the DT from one patient to another. Given the scope of the project, additional components need to be developed with sufficient support working with the community, and a path forward laid out in the project's blueprint.

### Project 5: virtual cancer digital twin approaches

#### Project overview: lead institution—University of Massachusetts, Amherst

Since each cancer has its own unique characteristics, each one can respond differently to the same treatments. Therefore, the creation of a digital twin (DT) of cancer can assist us in predicting the evolution of each cancer through computational modeling and finding the best treatment option for each patient. For each patient, the CPDT receives its information as input and predicts the evolution of their cancer. The CPDT will assist clinicians in the early detection of aggressive tumors and guide them to conduct timely surveillance, data collection, and choose appropriate treatments.

To reach this goal, it is proposed to take advantage of new advances in computational approaches and combine mechanistic, machine learning, and stochastic modeling approaches to create “My Virtual Cancer,” a CPDT platform. The team continues to develop a CPDT, which provides an *in-vivo* patient-specific experience to visualize the evolution of the disease of a given cancer patient based on the individual's disease characteristics. Users of this CPDT can visualize the evolution of a given cancer and its impact on other organs in the absence or presence of targeted therapies. This CPDT begins by using the patient's initial data, then suggests the time and type of new data be collected and updates itself accordingly.

This CPDT is based on the integration of data-driven mechanistic, machine learning, and stochastic agent-based models; all these models give constant feedback to each other to improve this dynamic CPDT. The proposed data-driven mechanistic model, which is a combination of biochemistry, biophysics, and PK-PD models, uses the compartmental-based scheme of quantitative systems pharmacology (QSP) modeling approach to model the entire body (26–28) QSP modeling is one of the main computational approaches used to discover, test, and predict the dose-exposure response. One of the main challenges of the QSP modeling is parameter estimation; parameters are calibrated using the data that are often assembled from disparate sources rather than a single curated data set (29, 30). As a result, they cannot be easily validated or used for personalized treatments. To establish a personalized CPDT, the team uses patient-specific data for parameter estimations, sensitivity analysis, and uncertainty quantification. For the parameter estimation, the approach considers the parameters as unknowns and determines them in the QSP model using state-of-the-art topology optimization methods. The team then performs a multi-dimensional sensitivity analysis and uncertainty quantification on the QSP model to find a set of critical interactions and predict the intervals of confidence.

The team analyzed 1,218 primary breast tumors in Breast Cancer (BRCA) data sets of the TCGA project (31) and 1,904 tumors in Molecular Taxonomy of Breast Cancer International Consortium (METABRIC) data (32). This was followed by a digital cytometry method on gene expression profiles of primary breast tumors to characterize the tumors' immune profiles and estimate the values of the mathematical model's variables to create a data-driven ordinary differential equation (ODE) model for human breast tumors (33). The team found that there are five distinct immune patterns of human breast tumors and investigated the dynamics of each of these immune patterns. The team also developed a data-driven ODE model for mice breast tumors (34) using the PyMT mice RNA-seq data (35) and extended it to a PDE model, which considers the observed spatial locations of key players in the mice breast tumors (36), using collaborator's data to document the differences between mice and human tumors and validate the model on mice data. The results of these models emphasize the importance of modeling cells' locations and separate parameter estimations for humans and mice.

#### Observations and future efforts

One of the main challenges of mathematical modeling of cancer is the lack of data, particularly time-course human multimodal data, for more reliable parameter estimation and validation. Future support is needed to be able to address some of the important limitations of current mathematical models by integrating available multi-modal patients' data and collaborating with biologists to estimate some of the parameters and validate the results. There are a limited number of computational models for rare cancer types, while patients with these cancers have mostly poor prognoses. For example, there are not any identifiable mathematical models for uveal melanoma (UM), and there is very limited publicly available data on UM. For the next phase of “My Virtual Cancer,” the team aims to (a) gather multi-modal patient data for UM and breast cancer; (b) analyze and merge different data types of both UM and breast cancer; (c) create a database with an API; (d) estimate the mechanical parameters of the mathematical model by performing some experiments; (e) improve mathematical models using pathology and MRI images; (f) perform parameter estimation using topology optimization; and (g) validate the model's predictions in mice and human and document the differences between them.

## Discussion

In terms of approaches, the five CPDT projects span a broad range of starting points and emphases. One variation among the projects is the selection of the type of cancer to pursue, involving pancreatic cancer, melanoma, NSCLC, and breast cancer. The projects also varied in their approaches with some incorporating mechanistic approaches, while others employed agent-based approaches. Not surprisingly, all used a form of AI, machine learning or data-driven approaches, albeit at different levels in which some used AI for model development while others used data-driven approaches for analysis of results or selecting the best models for individual patients.

The five exploratory projects have several recurring themes that share and underpin the need for an expanded community effort. First, the concept of a common framework, also emphasized by Wu et al. (5), underscores the importance of cooperative efforts and collaborations to advance the state of cancer patient digital twins. Secondly, while progress has been made within these projects, the reports of these efforts reinforce the critical need for additional patient-specific longitudinal data, particularly across populations that are representative of the community the CPDT is anticipated to support. Relatedly, and as expected, these data must also be multimodal, multiscale, and extensible to support the multiple levels of modeling and coherence necessary to achieve the CPDT.

These themes are not surprising, they were among the several challenges facing the development of CPDTs shared previously (1). Nonetheless, the five projects all demonstrated that biological and clinical domain knowledge, machine intelligent-driven analysis of large-scale multimodal datasets, and mechanistic modeling can be merged to create modular, reusable frameworks for CPDTs. Creative uses of artificial intelligence and large-scale model exploration for HPC resources have the potential to “shrink” seemingly intractable model calibration and featurization challenges into simpler problems that can be addressed by modern data assimilation techniques. As the community continues to explore and combined approaches, CPDTs will begin the march from science fiction to clinical reality.

The early outcomes of the CPDT pilot projects as well as the results from other CPDT efforts provide a sense of real promise for the future of the CPDT. As is evident, there are many potential future efforts identified within each of the five pilot projects, as well as future efforts that would span projects and other efforts involving the broader community. What is clear is that growing the CPDT community is among the highest priorities in realizing the potential of the CPDT and is the key focus for future efforts shared here.

The NCI-DOE Collaboration is committed to bringing together an ever-expanding and diverse community of interdisciplinary scientists across career stages from public and private organizations, such as the participants in the cancer patient digital twin projects. Focus areas emanate from a growing transdisciplinary community, termed the Envisioning Computational Innovations in Cancer Challenges (ECICC) community^6^. Since 2019, the ECICC community has engaged in collaborative activities with academia and other outside organizations across cancer, HPC and AI. There are over 200 members from multiple relevant disciplines, organizations and career stages registered through an NCI Hub site. ECICC has hosted numerous interactive events that have led to new partnerships and research projects at the intersection of cancer research and computational science/AI. For example, the recent report on predictive radiation oncology (6) was created by the ECICC community, and now serves as a resource to the broader cancer research community. The ECICC site includes a dedicated CPDT resource area to serve as a hub for growing the CPDT community.

Future research projects in digital twin technologies, predictive radiation oncology and other grand cancer challenges are expected to engage broader communities and lead to disease and intervention-specific models and simulations, using mathematical, active learning, and ensemble model approaches for cancer and other areas of biomedical research. Additional, robust initiatives are underway to expand the community and increase opportunities for interdisciplinary, cross-organizational research projects. Broader engagement with the research community, new resources and collaborative research opportunities developed by the NCI-DOE Collaboration are shaping the future of predictive oncology, drug discovery, and clinical applications. To join this dynamic community or for more information, contact ECICC_Community@nih.gov*.*

## Data Availability

The original contributions presented in the study are included in the article/Supplementary Material, further inquiries can be directed to the corresponding author/s.

## Footnotes

^1^Online reference: https://obamawhitehouse.archives.gov/precision-medicine accessed July 18, 2022.

^2^Online reference: https://www.digitaltwinconsortium.org/initiatives/the-definition-of-a-digital-twin/ on July 18, 2022.

^3^Online reference: https://www.nitrd.gov/nsci/ accessed July 18, 2022. The NSCI was launched by Executive Order (EO) 13702 in July 2015 to advance U.S. leadership in high performance computing (HPC).

^4^https://www.intel.com/content/www/us/en/government/predictive-oncology-and-computing.html, accessed July 18, 2022.

^5^https://events.cancer.gov/cbiit/dtwin2020, accessed July 18, 2022.

^6^The Envisioning Computational Innovations and Cancer Challenges website is located at https://ncihub.org/groups/cicc/overview.
